# Immunoprotection Provided by Salivary and Intestinal Protein-Based Antigens Against the Ixodid Tick *Amblyomma sculptum*

**DOI:** 10.3390/vaccines13020136

**Published:** 2025-01-28

**Authors:** Ulisses A. Natividade, Jessica F. Abreu, Izabela C. T. Ribeiro, Adalberto A. Pereira Filho, Augusto V. Silva, Helen S. Ribeiro, Rodolfo C. Giunchetti, Mauricio R. V. Sant’Anna, Nelder F. Gontijo, Marcos H. Pereira, Ricardo N. Araujo

**Affiliations:** 1 Laboratory of Hematophagous Arthropods, Department of Parasitology, Institute of Biological Sciences, Universidade Federal de Minas Gerais, Belo Horizonte 31270-901, MG, Brazil; 2 Laboratory of Cell-Cell Interactions, Department of Morphology, Universidade Federal de Minas Gerais, Belo Horizonte 31270-901, MG, Brazil; 3 Laboratory of Hematophagous Insects Physiology, Department of Parasitology, Institute of Biological Sciences, Universidade Federal de Minas Gerais, Belo Horizonte 31270-901, MG, Brazil; 4 Instituto Nacional de Ciência e Tecnologia em Entomologia Molecular, Universidade Federal do Rio de Janeiro, Rio de Janeiro 21941-853, RJ, Brazil

**Keywords:** anti-tick vaccine, tick control, saliva, intestine, immune response, vaccine efficacy

## Abstract

**Background/Objectives:** *Amblyomma sculptum* is among the most dangerous ticks in South America, as it is the species most associated with humans and is the main vector of *Rickettsia rickettsii*. In the face of the problems related to tick control based on chemical acaricides, vaccines emerge as a promising method. In previous works, three salivary recombinant proteins (rAs8.9kDa, rAsKunitz, and rAsBasicTail) and one protein based on intestinal immunogenic regions (rAsChimera) were described with 59 to 92% vaccine efficacy against *A. sculptum* females. Here, we evaluate novel vaccine formulations containing binary or multiple combinations of the antigens rAs8.9kDa, rAsKunitz, rAsBasicTail, and rAsChimera against the three instars of the tick. **Methods:** A control group of mice was immunized with adjuvant alone (aluminum hydroxide gel) and compared to five groups immunized with formulations containing two, three, or four of the antigens. **Results:** The formulations were safe, with no significant alterations to host behavior and hematological or biochemical parameters. Immunizations induced a significant increase in the CD19^+^ B lymphocyte percentage in all groups, but no difference was seen for CD8^+^ and CD4^+^ T lymphocytes or CD14^+^ monocytes. The best protection was observed for the formulations containing two antigens, which reached above 98% efficacy, while the groups containing three or four antigens presented 92.7 and 94.4% efficacy, respectively. **Conclusions:** All antigen combinations were promising as vaccine formulations against *A. sculptum*. The formulation containing rAs8.9kDa and rAsChimera showed the best efficacy and should be focused on in further experiments.

## 1. Introduction

*Amblyomma cajennense sensu lato* ticks have a wide distribution across the American continent, spanning from the southern United States to northern Argentina [1]. Among the six species of the complex, *Amblyomma sculptum* Berlese, 1888 is the most important in South America, as it is found in the most populated areas, is the primary tick associated with humans [2,3,4], and is the main vector of the bacteria *Rickettsia rickettsii*, the causative agent of the Brazilian spotted fever [5,6]. The species has been reported in Argentina, Bolivia, Brazil, and Paraguay, mainly associated with the biomes in Cerrado and the degraded areas of the Atlantic rainforest [3]. Horses, capybaras, and tapirs are among the primary hosts of *A. sculptum*, but several other domestic and wild species of hosts, including mammals, birds, and reptiles, have been found parasitized, especially by immature instars, which are considered to have low host specificity [7]. Additionally, *A. sculptum* is associated with most of the human cases of Brazilian spotted fever, caused by the bacteria *Rickettsia rickettsii* [5,6]. The bacteria can be transmitted by any of its instars due to the occurrence of transovarial transmission [8].

Tick control is still mainly based on chemical acaricides [9,10], a method that has aroused concerns due to the possibility of contaminating the environment, products of animal origin, and workers dealing with animals [11]. The use of acaricides can also select resistant tick populations, and this concern is also shared for *A. sculptum* [12,13].

Developing novel and more efficient ways to control *A. sculptum* may help solve or minimize the problems generated by chemical acaricide treatments. Anti-tick vaccines are suitable alternatives, as they are more environmentally friendly and may be used alone or combined with acaricides or other control methods. The use of vaccines may contribute significantly to the reduction of ticks on hosts and also in the environment, thus reducing the occurrence of diseases caused by tick-borne pathogens.

Few studies have been carried out to identify antigens and develop vaccines against ticks of the *A. cajennense* complex. The BM86 antigen, the basis of commercial vaccines against the cattle tick *Rhipicephalus microplus*, was found to be ineffective against *A. cajennense s. l.* [14], while the P0 antigen, initially also isolated from *R. microplus*, demonstrated 56% efficacy against *A. mixtrum*, another species of the *A. cajennense* complex [15]. Additionally, proteins associated with apoptosis inhibition were investigated as possible targets against *A. sculptum* using RNA interference, showing promising results as potential vaccine candidates, but vaccination trials still need to be carried out [16]. Recently, Costa et al. [17] identified three multifunctional *A. sculptum* salivary proteins, named AsKunitz, As8.9kDa and AsBasicTail. In vaccination trials conducted on mice, these proteins demonstrated efficacies of 85.3, 92.8, and 59.4% against female ticks. Furthermore, a chimeric recombinant protein constructed from a sequence of 22 immunogenic regions from gut molecules (named rAsChimera) showed an efficacy of 80.8% against females [18].

The possibility of creating anti-tick vaccines has been explored since earlier studies of tick infestation showed that hosts can acquire effective immune responses against ticks after initial parasitism, as is commonly seen for several *Bos indicus* breeds [19]. Studies on the BM86 antigen have shown that antibodies play an important role in the protection, either by IgG alone or with the aid of the complement system of the host. Antibodies induce damage to the gut epithelium, reducing tick viability, hematophagy capacity, and reproductive performance [20,21,22,23]. Therefore, the present work explores the hypothesis that enhancing the attack on the gut epithelium by the host complement system may lead to higher efficacies of anti-tick vaccines. For this, the present work describes the results of vaccine trials using formulations containing binary (rAs8.9kDa and rAsChimera, rAsBasicTail and rAsChimera, or rAsKunitz and rAsChimera) or multiple (rAs8.9kDa, rAsKunitz, and rAsChimera or rAs8.9kDa, rAsKunitz, rAsBasicTail, and rAsChimera) combinations of antigens based on gut surface and anti-complement proteins [17,18], detailing the vaccines’ safety, immunological assessments, and the effect on the parameters used to calculate vaccine efficacy.

## 2. Materials and Methods

### 2.1. Experimental Ticks and Ethical Statement

Ticks used in the experiments were obtained from an *A. sculptum* colony kept at the Laboratory of Hematophagous Arthropods, UFMG. Ticks were maintained in a BOD incubator under controlled conditions of temperature (28 ± 2 °C) and humidity (85 ± 5%), and were fed on Swiss mice using feeding chambers according to the methodology described by Bouchard and Wikel [24]. All ticks used were aged between 20 and 40 days after hatching or molting. The Ethics Committee on the Use of Animals at the Universidade Federal de Minas Gerais (CEUA/UFMG) approved the maintenance of our tick colony and the vaccine experiments (protocols 06/2020 and 103/2017).

### 2.2. Vaccine Formulations and Experimental Groups

Four recombinant antigens identified in previous works were evaluated in the vaccine trials: rAs8.9kDa—a 13 kDa protein containing a von Willebrand factor (vWF)-type C domain; rAsKunitz—a 10.9 kDa protein containing a Kunitz_BPTI-type domain; rAsBasictail—an 18.9 kDa protein that contains several lysine residues in the carboxy-terminal region; and rAsChimera—a 48.5 kDa protein that contains 22 immunogenic regions similar to those found in the seven intestinal proteins of *A. sculptum* [17,18]. The sequence of each antigen and the production of the recombinant proteins in *Escherichia coli* BL21 were previously described [17,18]. Briefly, sequences were cloned into pET28a-TEV vectors and transformed into bacteria by heat shock. Purification was carried out using His Trap^®^ Hp affinity columns (GE Healthcare Life Sciences, Chicago, IL, USA) followed by performing affinity chromatography with an AKTA-prime plus^®^ (GE Healthcare Life Sciences). The samples were evaluated using SDS-PAGE and had their protein content measured [25]. Expression of the recombinants was confirmed by western blotting and plasmid sequencing.

The recombinants were used to produce five vaccine formulations containing five µg of each protein and 0.1 mg of aluminum hydroxide gel (Sigma–Aldrich, St. Louis, MO, USA.) as an adjuvant, which was dissolved in a final volume of 100 µL of sterile PBS (137 mM NaCl, 10 mM Na_2_HPO_4_, 2.7 mM KCl, pH 7.4). The vaccinated groups and the recombinant proteins used in each of the formulations were: V1—rAs8.9kDa and rAsChimera, V2—rAsBasicTail and rAsChimera, V3—rAsKunitz and rAsChimera, V4—rAs8.9kDa, rAsKunitz and rAsChimera, and V5—rAs8.9kDa, rAsKunitz, rAsBasicTail, and rAsChimera. The control group (Ct) was injected with 0.1 mg of adjuvant (aluminum hydroxide gel) in PBS. The vaccinated groups were challenged with larvae, nymphs, and adult couples (one female and one male) of *A. sculptum*.

### 2.3. Immunization of the Experimental Groups

**Figure 1:**
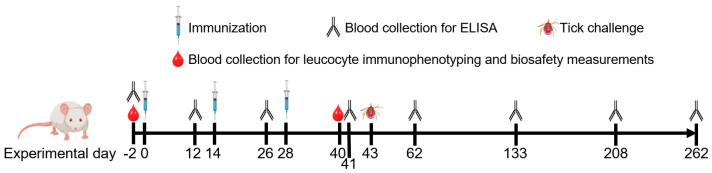
Schematic diagram of the immunization, blood collections, and tick challenge.

The vaccination experiments were performed using 4-to-6-week-old male Swiss mice obtained at the UFMG’s Bioterism Centerterism (CEBio). The animals were maintained in a 12:12-hour light–dark cycle and received rodent food and water ad libitum. Three doses of the formulations were applied to each animal subcutaneously on the animal’s back at 14-day intervals (Figure 1). Individual blood samples were collected by puncturing the caudal lateral vein two days before immunizations (D-2) and at different times after vaccination had started, up until day 262 (D262) (Figure 1). Three sets of mouse groups were used and challenged with each tick instar. Groups challenged with larvae and nymphs contained five mice each, while ten mice were used for groups of adults. Ticks that fed on animals that died or showed any health or behavioral alterations during the challenge were removed from the analysis.

### 2.4. Behavioral Analysis and Immunobiological Safety

Vaccination safety was clinically assessed by macroscopic analysis of the inoculation site to check for the presence of nodules or wounds within 72 h following each injection. In addition, the animals’ behavior was checked daily to identify any possible alterations.

Immunobiological safety was also assessed by checking the hematological and serum biochemical parameters on D-2 and day 40 (D40) of five animals in each group. This involved collecting approximately 50 μL of blood in 0.5 mL tubes containing EDTA (Vacuplast, Cotia, SP, Brazil), which was then used for erythrocytic series measurements (erythrocytes, hemoglobin, and hematocrit) and total leukocytes measurements using an Abacus Analyzer (Celer Biotecnologia, Belo Horizonte, MG, Bazil). The biochemical profile (urea, creatinine, aspartate aminotransferase (AST), and alanine aminotransferase (ALT)) was also evaluated. For this, approximately 50 μL of blood was collected in a 1.5 mL microcentrifuge tube, which was kept resting in room temperature for 30 min, was centrifuged at 600× *g* for 10 min, and then the entire serum volume was collected and analyzed using a Cobas Mira Biochemical Analyzer (Roche, Basel, Switzerland).

### 2.5. Phenotypic Profile of the Lymphocyte Populations

The lymphocyte phenotypic profile of the immunized animals was evaluated by flow cytometry using blood collected from five animals in each group. Aliquots containing 50 µL of mouse blood were transferred to 5 mL polystyrene tubes (BD Falcon round-bottom polystyrene tubes) containing 20 µL of anti-CD3-Percep-Cy5 antibodies (clone 145-2C11, eBioscience, Thermo Fisher Scientific), anti-CD4-FITC (clone RM4-5, eBioscience, Thermo Fisher Scientific, Waltham, MA, USA), and anti-CD8-APC (clone 53-6.7, eBioscience, Thermo Fisher Scientific). An additional 50 μL was transferred to a polystyrene tube containing the anti-CD19-FITC antibody (clone MB19-1, eBioscience, Thermo Fisher Scientific), and an extra 50 μL was transferred to a polystyrene tube containing the anti-CD14-APC antibody (clone Sa2-8, eBioscience, Thermo Fisher Scientific). The tubes were homogenized by vortexing and incubated for 30 min in the dark. After incubation, 2 mL (1 mL at a time) of Billing Dog solution (Facs lysing solution, Becton Dickinson) was added to the vortex and incubated for 10 min in the dark. After incubation, the lysis reaction was stopped by adding 1 mL of PBS wash (PBS containing 0.5% bovine serum albumin (BSA) and 0.1% sodium azide, pH 7.2), and samples were centrifuged at 600× *g* for 7 min at 18 °C. After centrifugation, the supernatant was discarded, and the pellet was washed using 2 mL of PBS-wash. The pellet was then resuspended in 200 μL of Macs Facs Fix fixative solution (10.0 g/L paraformaldehyde, 10.2 g/L sodium cacodylate, and 6.65 g/L sodium chloride) for reading on a FACSCalibur flow cytometer (Becton Dickinson) with acquisition of 30,000 events/tube. The software FlowJo 10.4 (Tree Star) was used to analyze cellular immunophenotyping. Total lymphocytes were identified by gating on the FSC versus SSC plot (Supplementary Materials—Figure S1A). CD4^+^ and CD8^+^ T cells were selected based on their co-expression with the CD3 T cell marker (CD4^+^CD3^+^ and CD8^+^CD3^+^, respectively) within the total lymphocytes gate (Supplementary Materials—Figure S1B,C). B cells were identified as CD19^+^ cells within the total lymphocyte gate (Supplementary Materials—Figure S1D). The results for CD4^+^CD3^+^, CD8^+^CD3^+^, and CD19^+^ were expressed as percentages of positive cells within the total lymphocyte gate. Monocytes were characterized by CD14^+^ expression and intermediate granularity (Supplementary Materials—Figure S1E).

### 2.6. Humoral Response

To evaluate the production of antigen-specific antibodies, 50 µL of individual blood samples were collected from 13 animals of each group and the serum was obtained as described above. Antigen-specific IgG levels for each of the vaccine targets were determined individually in the hosts’ serum through enzyme-linked immunosorbent assays (ELISAs) using 0.5 μg of each purified antigen in 50 μL of carbonate buffer/well in 96-well ELISA plates (COSTAR^®^ 3590 high binding). After incubation at 4 °C in a humid chamber overnight, the plates were washed and blocked for 2 h at 37 °C with agitation with a blocking solution (PBS containing 2% casein). After three washes with PBS-Tween (PBS with 0.05% Tween 20), the plates were incubated for 1 h with shaking at 37 °C and with sera at a 1:320 dilution in PBS-Tween, and after further washing, incubated with anti-mouse IgG conjugated to peroxidase (Sigma–Aldrich^®^) at a 1:10,000 dilution with PBS-Tween. Results were visualized using 100 mM of phosphate–citrate buffer (pH 5.0) that contained 0.025% H_2_O_2_ and 0.01% o-phenylenediamine. Reactions were stopped with the addition of 200 μL of 12.5% H_2_SO_4_, and the absorbance was measured at 492 nm in an ELISA reader (VERSAmax^®^, Molecular Devises, San Jose, CA, USA) with the SoftMax^®^ pro 3.0 software (Molecular Devices).

### 2.7. Tick Challenge and Assessment of Efficacy

The tick challenge was performed 15 days after the last immunization (day 43). Each mouse was infested with 50 larvae, 10 nymphs, or one male and one female using feeding chambers [24]. Several feeding and development parameters were measured, such as feeding period, final weight, percentage of feeding, percentage of molt, and mortality. Female reproductive parameters, such as fertile females (females that laid eggs), egg mass, and larvae hatching, were also evaluated. Vaccine efficacy, considering the parameters assessed from larvae, nymphs, and females, was calculated according to Aguirre et al. [26] using the formula E(%) = 100 × [1 − (RL × VL × RN × VN × RA × OA × FE)] where: RL is the effect of vaccination on larvae recovery = RLvaccinated/RLcontrol; RN is the effect of vaccination on nymph recovery = RNv/RNc; RA is the effect of vaccination on adult female recovery = RAv/Rac; VL is the effect on larvae viability = VLv/VLc; VN is the effect on nymph viability = VNv/VNc; OA is the effect on female oviposition, which is the ratio between the average weight of the egg mass laid by females in the vaccinated (OAv) and control (OAc) groups = OAv/OAc; and FE is the effect on egg fertility, which is the ratio between the average hatchability (%) of eggs laid by females in the vaccinated (FEv) and control (FEc) groups = FEv/FEc.

### 2.8. Statistical Analysis

Data were transferred to GraphPad Prism 7.0 software for statistical analysis. Data are represented by means ± standard errors or means ± standard deviations, and each tick was considered as one individual replicate. Data normality was assessed using the Kolmogorov–Smirnov test. The analysis of variance (ANOVA) test was performed for data with a normal distribution, followed by Tukey’s or Dunnett’s post-test. Non-normally distributed data were analyzed using the Kruskal–Wallis test and Dunn’s post-test. The significance level considered was *p* < 0.05.

## 3. Results

### 3.1. Immunobiological Safety and Harm Analysis

Immunizations induced no adverse reactions in mice in the experimental groups. No nodules, papules, or ulcers were seen at the inoculum sites, and no animal behavioral changes were observed. Biochemical and hematological parameters were assessed in all experimental groups at D-2 and D40, and no major alterations that indicated an impact on homeostasis were induced by the vaccinations (Supplementary Materials—Tables S1 and S2). The alterations observed were a significant increase (*p* < 0.05) between D-2 and D40 in the AST levels of groups V1, V2, and V5, and a significant decrease (*p* < 0.05) in MCHC levels in group V3.

### 3.2. Leukocyte Immunophenotyping

**Figure 2:**
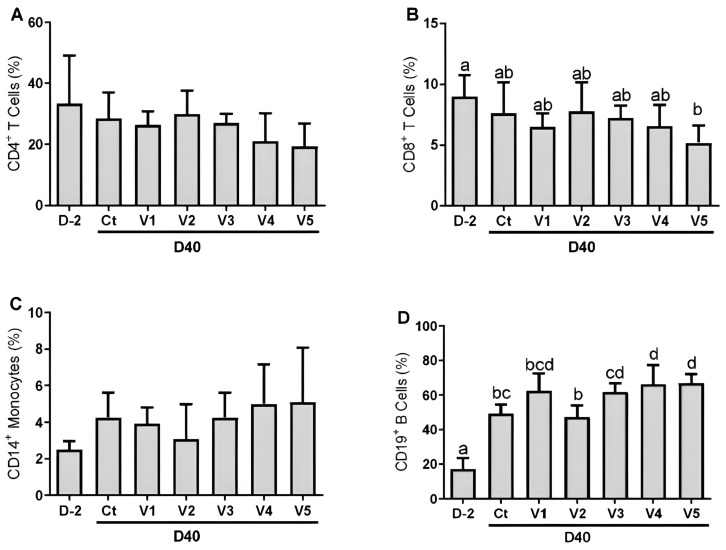
Comparative analysis of leukocytes by flow cytometry using whole blood before immunization (D-2) and 12 days after the last dose (D40) in the control (Ct) and vaccinated (V1–V5) groups. The leukocytes evaluated were CD4^+^CD3^+^ (**A**), CD8^+^CD3^+^ (**B**), and CD14^+^ (**C**), and CD14^+^ (**D**). The vaccine formulations contained the antigens rAs8.9kDa (8.9), rAsBasicTail (BT), rAsKunitz (Kn), and rAsChimera (Chi). Experimental groups: Ct—control (adjuvant); V1—8.9+Chi; V2—Bt+Ch; V3—Kun+Ch; V4—Kun+8.9+Ch; V5—Kun+Bt+8.9+Ch. Data are shown as mean ± SD. Blood samples collected from five animals in each group were used in the analysis. Statistical analysis: ANOVA and Tukey’s multiple comparison test; different letters indicate a significant difference (*p* < 0.05).

Analysis of leucocytes from experimental mice showed no significant differences (*p* > 0.05) in CD4^+^ T cells and CD14^+^ monocytes between D-2 and D40 or between control and vaccinated groups at D40 (Figure 2A,C). The percentage of CD8^+^ T cells was similar (*p* > 0.05) for all comparisons, except for a significant decrease (*p* < 0.05) seen in group V5 at D40 in comparison to D-2 (Figure 2B).

CD19^+^ B cells were affected by the immunizations. All groups saw significant increases (*p* < 0.05) in the percentage of CD19^+^ B cells from D-2 to D40. There were significant differences (*p* < 0.05) between groups at day 40, as groups V4 and V5 had a significantly higher (*p* < 0.05) percentage of cells in comparison to controls, while group V2 had a significantly lower percentage of cells in comparison to V3, V4, and V5 (Figure 2D).

### 3.3. Humoral Response

**Figure 3:**
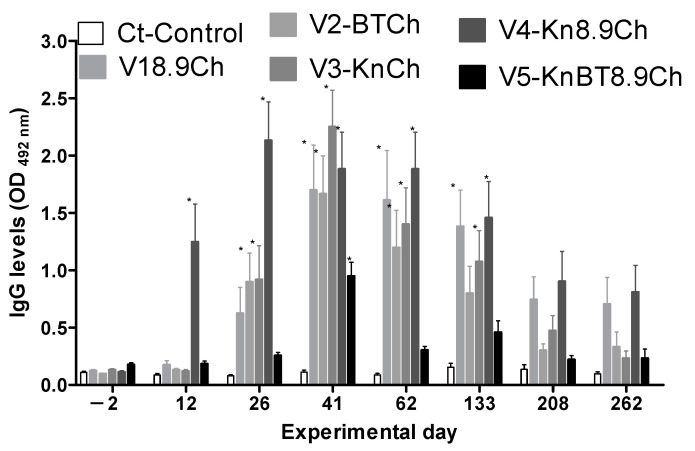
Humoral response produced against the antigens used for the immunization of mice. Total IgG levels were measured by ELISA; plates were sensitized with all four antigens combined (rAs8.9kDa (8.9), rAsBasicTail (BT), rAsKunitz (Kn), and rAsChimera (Ch)). Experimental groups: Ct—control (adjuvant); V1—8.9+Chi; V2—Bt+Ch; V3—Kun+Ch; V4—Kun+8.9+Ch; and V5—Kun+Bt+8.9+Ch. Data are represented by mean ± SE. Blood collected from 13 animals in each group was used in the analysis. Statistical analysis: two-way ANOVA and Dunnett’s multiple comparison test; asterisks indicate a significant difference compared to Ct (*p* < 0.05).

**Figure 4:**
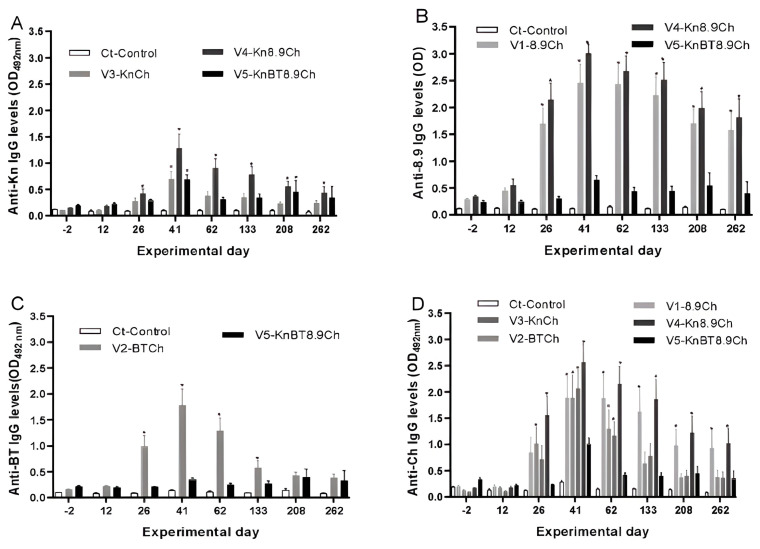
Antigen-specific IgG levels of mice immunized with the rAsKunitz, rAs8.9kDa, rAsBasicTail, and AsChimera combinations. Total IgG levels were measured by ELISA; plates were sensitized with the four antigens individually as follows: (**A**) rAsKunitz (Kn), (**B**) rAs8.9kDa (8.9), (**C**) rAsBasicTail (BT), and (**D**) rAsChimera (Ch). Experimental groups: Ct—control (adjuvant); V1—8.9+Chi; V2—Bt+Ch; V3—Kun+Ch; V4—Kun+8.9+Ch; and V5—Kun+Bt+8.9+Ch. Data are represented by mean ± SE. Blood collected from five animals in each group was used in the analysis. Statistical analysis: two-way ANOVA and Dunnet’s multiple comparison test; asterisks indicate significant differences compared to the control (*p* < 0.05).

The antigen-specific IgG levels significantly increased in all vaccinated groups after immunizations. Overall, IgG levels were significantly higher on D26 (after the second immunization) and reached a peak on D41 (after the third immunization), followed by a gradual reduction until the end of the experiment (Figure 3 and Figure 4).

When the IgG levels against all four antigens were analyzed together, the profiles and levels were relatively similar between groups V1–4, while group V5 had significantly lower IgG levels throughout the whole experimental period (Figure 3). 

Analysis of the IgG levels against each antigen confirmed that the formulation used in V5 induced lower IgG levels against all four antigens (Figure 4). Concerning the other groups, overall results showed that rAs8.9kDa had the higher antiserum-specific IgG levels (Figure 4B), followed by rAsChimera (Figure 4D) and rAsBasicTail (Figure 4C), while rAsKunitz had the lowest levels, which were, nevertheless, significantly (*p* < 0.05) higher than the control’s (Figure 4A). Interestingly, rAs8.9kDa antiserum levels remained higher until D262 in groups V1 and V4, while specific IgG levels decreased earlier for all other antigens (Figure 4).

### 3.4. Tick Challenge on Immunized Mice

**Figure 5:**
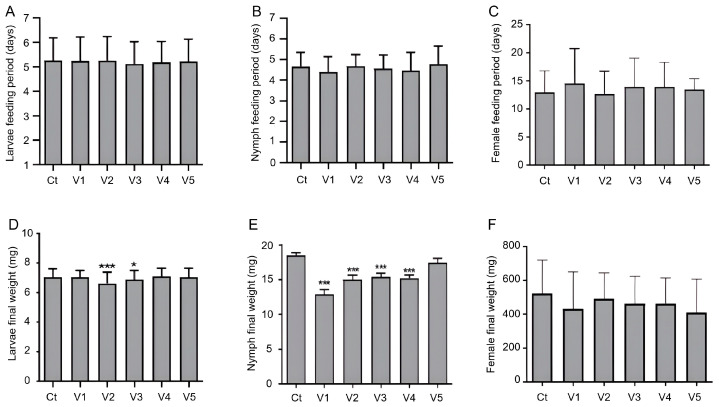
Feeding period and final weight of *A. sculptum* fed on mice immunized with the antigens rAs8.9kDa (8.9), rAsBasicTail (BT), rAsKunitz (Kn), and AsChimera (Ch). Parameters were measured from larvae (**A**,**D**), nymphs (**B**,**E**) and females (**C**,**F**). Experimental groups: Ct—control (adjuvant); V1—8.9+Ch; V2—Bt+Ch; V3—Kn+Ch; V4—Kn+8.9+Ch; and V5—Kn+Bt+8.9+Ch. Data are shown as mean ± SD. Statistical analysis: Kruskal–Wallis test and Dunn’s post-test; asterisks indicate significant differences to the control: * *p* < 0.05, *** *p* < 0.001.

**Figure 6:**
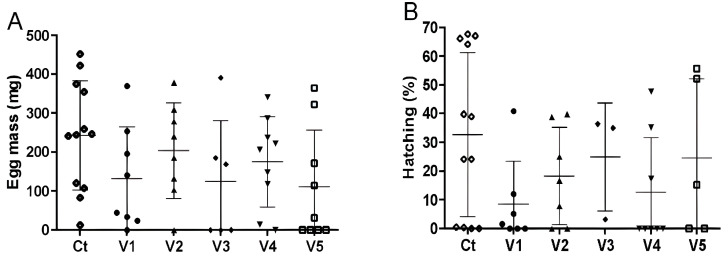
Reproductive parameters of *A. sculptum* females fed on mice immunized with rAs8.9kDa (8.9), rAsBasicTail (BT), rAsKunitz (Kn), and AsChimera (Ch) combinations. Reproductive parameters: (**A**) egg mass laid by females and (**B**) percent of hatching larvae. Experimental groups: Ct—control (adjuvant); V1—8.9+Ch; V2—Bt+Ch; V3—Kun+Ch; V4—Kun+8.9+Ch; V5—Kun+Bt+8.9+Ch. Data are shown as mean ± SD. Statistical analysis: one-way ANOVA and Dunn’s multiple comparison test; no significant differences were observed between groups.

The study revealed that the three tick instars had some parameters affected when fed on immunized mice. In general, molting was the most impacted parameter of immature instars, while females experienced fertility-related effects (Table 1 and Figure 5 and Figure 6). Upon detailed evaluation, larvae showed no issues in feeding on immunized mice (Figure 5A), but ticks from groups V2 and V3 had significantly (*p* < 0.05) lower final weights in comparison to controls (Figure 5D). The most considerable differences were observed in larvae recovery and molting, with the percentage of larvae able to feed and reach the nymph instar being less than 15% in groups V1, V2, and V3 (Table 1).

For nymphs, the feeding period was also not affected by immunizations (Figure 5B); however, the final weight was significantly reduced in groups V1 and V4 (Figure 5E), and molt was reduced to less than 20% in groups V1, V2, and V3 (Table 1). Similar to larvae, groups V1 and V3 were the most affected, as total mortality was higher than 80% for V2 and V3, and 92% for V1 (Table 1).

During the adult challenge, two mice died (from groups V1 and V2) and their data were removed from the analysis. Female ticks had no significant differences (*p* > 0.05) compared to the controls in their feeding period and final weight (Figure 5C,F), as well as in the egg mass (Figure 6A) and percent of hatching larvae (Figure 6B). However, the percentage of fed females was reduced, mainly in groups V3 and V4, as well as the percentage of fertile females in groups V3 and V5 (Table 1).

The feeding and development parameters of all the instars, along with the female reproductive parameters, were used to calculate the overall efficacies of each vaccine formulation compared to the control. Three of the four formulations had an efficacy higher than 90%, with the best results observed in groups V1 and V3, whose efficacies were higher than 99% (Table 2).

## 4. Discussion

The present work is part of a series of studies conducted by Costa et al. [17,18], and adds novel information to the search for a vaccine against the tick *A. sculptum*. Novel formulations using the antigens rAsKunitz, rAs8.9kDa, rAsBasicTail, and rAsChimera were tested, and additional aspects of the immune response induced by immunizations were assessed, as well as the safety of the inoculums and the leukocyte phenotypes. Also, the tick challenge was carried out with all three tick instars used for efficacy calculations. The results confirmed the potential of the proteins rAs8.9kDa, rAsKunitz, rAsBasicTail, and rAsChimera to become antigens against *A. sculptum*.

The vaccine formulations were constructed based on the possible mechanism of action previously described for the *R. microplus* intestinal hidden antigen BM86, where protection from ticks was correlated with the level of antibodies against midgut membrane antigens and the lytic effect caused by the host’s blood complement system [20,21,22,23]. As the three salivary antigens used in the present work are inhibitors of the complement system [17], we hypothesize that anti-rAsChimera antibodies could activate the classical pathway of the host’s blood complement system by binding to epitopes of proteins on the intestinal epithelium’s surface, and antibodies against these salivary proteins would prevent the complement inhibition promoted by those molecules. Those antibody features would induce a strong complement attack on the tick’s gut epithelium. Therefore, all vaccine formulations contained an antigen based on intestinal immunogenic regions (rAsChimera) plus one, two, or three salivary antigens.

For insects, complement inhibition at the intestinal level is important to avoid damage to the epithelium [27,28], and a similar phenomenon may occur with ticks. The effector mechanisms of the formulations tested here still need to be elucidated, but the data on high mortality in immature stages before molting (Table 1) is compatible with our hypothesis, which involves antibodies and the complement system.

Vaccine formulations were safe, as no toxic effects were seen on the immunized animals either at the injection site or in the liver or kidney physiology. Hematological parameters were also not altered. This is an expected result, as the formulations do not contain any potentially harmful components. In addition, the adjuvant used was an aluminum compound, which is considered safe and has been used in humans and animals for several years [29].

Induction of the immune response was seen in all immunized groups. The leukocyte phenotyping showed that B cells had the most marked difference of the cell types analyzed, showing greater proliferation. However, the presence of the adjuvant was crucial since the control group also showed an increase from D-2 to D40, an expected effect of aluminum hydroxide [29]. A similarity in monocyte and CD3^+^ cells’ circulating levels was observed in a previous anti-tick vaccine trial [30] and is in line with an expected, more intense anti-tick immune response at the bite site instead of at the circulation level [31].

The most positive parameter of the immunizations was the increased IgG levels, an extremely desired response in anti-tick vaccines [20]. The inoculants given to all groups induced increased antigen-specific IgG levels, although some responded better than others. The IgG increase varied from about ninefold for anti-rAs8.9kDa IgG in group V4 to about threefold for anti-rKunitz IgG in group V3.

Overall, groups that contained the rAs8.9kDa antigen had the highest and longest-lasting IgG levels (Figure 4B), corroborating previous work that used the same antigen [17]. It is of note that group V4 had the earliest humoral response, with significantly higher antigen-specific IgG levels before the second immunization. The reason for such an increase remains unclear and should be further investigated. On the other hand, group V5 contained rAs8.9kDa in the formulation and showed the lowest antiserum IgG results. The amount of antigens in this formulation (20 µg/inoculum) impaired the higher elevation of antigen-specific IgG levels, which were significantly higher than controls only on D41. Indeed, the reduction in the production of specific antibodies with different antigen combinations has been discussed in the literature and may be a consequence of antigenic competition and immunotolerance, among other possibilities [32]. It is also of note that bacterial contaminants could have been present in the inoculums, as the recombinant proteins were produced in *E. coli*, and the presence of even low amounts of LPS or other bacterial contaminants could have influenced the antigen-specific humoral and cellular responses [33,34]. Such an occurrence could have impaired a higher IgG response in all groups, a possibility that is in line with the results from the V5 group, which was the one that contained the higher amounts of recombinants in the inoculum.

Notably, significantly higher IgG levels than controls were seen for most groups and proteins until D262. The inoculums were responsible for the increased IgG levels, but tick salivation during feeding can also induce specific antibody production [35]. The sequences of rAsKunitz, rAsBasicTail, and rAs8.9kDa are based on salivary exposed proteins, so tick feeding during the challenge may have helped maintain the specific IgG levels higher for extended periods. On the other hand, rAsChimera is based on concealed intestinal immunogenic regions, and no antigenic boost during feeding was expected [36]. Therefore, the results indicate that the inoculum stimulus alone was sufficient to maintain higher anti-rAsChimera IgG levels until D262 in groups V1 and V4.

The tick challenge showed that feeding time was not affected by immunizations, but the volume of blood ingested was reduced for larvae and nymphs. Other affected parameters were the number of ticks that completed feeding (especially for larvae and females), molting time, and female fertility. Those parameters contributed to overall vaccine efficacy, which was above 90% in three of the four groups and above 99% in three. Those efficacies were equivalent to the ones observed for the antigens rAsKunitz, rAsBasicTail, rAs8.9kDa, and rAsChimera, which achieved 59 to 92% against females and up to 100% against nymphs [17,18]. Efficacies were also higher than in other works that tested antigens against *A. cajennense s.l.*, where a BM86 formulation had no efficacy at all [14], and the P0 antigen was only 54% effective against *A. mixtrum* [15]. However, it is of note that the efficacy of the present work was calculated according to Aguirre et al. [26], which describes a formula used for three host ticks, applied here due to the challenges performed with all three tick instars. On the other hand, the efficacy of previous works with *A. cajennense s.l.* was calculated based only on adult challenges and used a formula adapted from one host tick [37] that considered only female data.

Thus, the results obtained in the present work, together with previous findings [17,18], are encouraging and suggest that further studies should focus on the combination of rAs8.9kDa with rAsChimera. To do this, those studies should provide a better characterization of the immune response induced by vaccination, especially if the protective response is indeed dependent on complement activation in the gut environment. If this is the case, the focus should be on the production of antibody subtypes that trigger the classical pathway of the complement system [38], whose levels should be measured and for which novel means of elevation should be developed. Also, further studies should test antigen protection in natural hosts, as efficacies obtained in laboratory models can be as high as 99% [39,40] but are normally less effective when tests are carried out with natural hosts [41,42,43,44,45,46]. For *A. sculptum*, the main targets are horses and capybaras, which are considered the primary domestic and wild hosts, respectively, and have been implicated as tick maintainers in most of the areas of reported transmission of *R. rickettsii* in Southeast Brazil [6]. As *A. sculptum* is a seasonal tick that needs one year to complete one generation in the natural environment [7], if the vaccine efficacies observed here are repeated in the natural hosts, a few years of vaccination would be enough to achieve effective environmental population control. This strategy would be advantageous for tick control in horses and capybaras, for which management imposes a significant impairment on urban tick control [47,48].

## 5. Conclusions

Results of the immunization and tick challenge trials indicate that all antigen formulations were safe for the hosts; four of them induced a humoral response with increased levels of antigen-specific IgG and three of the formulations achieved more than 99% vaccine efficacy in mice. In general, tick feeding parameters were not affected by the vaccinations, and the final weight was reduced only slightly in some groups of larvae and nymphs. However, the larvae and nymph viability after feeding, the egg production by females, and the percentage of eggs that hatched were considerably affected and were the tick parameters that contributed most to the vaccines’ efficacy. Efficacies obtained in the present work, in addition to previous results with the same antigens [17,18], indicate that the best vaccine formulation was the combination of rAs8.9kDa and rAsChimera, and this formulation should be applied in further studies to produce a viable vaccine against *A. sculptum*.

## 6. Patents

Patents resulting from the work reported in this manuscript: BR 13 2024 006304 9.

## Figures and Tables

**Table 1:** Feeding, development and reproductive parameters of *A. sculptum* fed on mice previously immunized with the rAs8.9kDa (8.9), rAsBasicTail (BT), rAsKunitz (Kn), and rAsChimera (Ch) combinations.

	Groups	Ct (Control)	V1 (8.9Ch)	V2 (BtCh)	V3 (KnCh)	V4 (Kn8.9Ch)	V5 (KnBt8.9Ch)
Parameter							
Larvae							
Fed (%) ^1^		98.0	66.4	71.6	82.6	93.8	97.5
Molted (%) ^2^		95.0	13.3	16.1	17.0	50.0	59.9
Mortality (%) ^3^		6.9	91.9	88.4	87.8	53.1	41.6
Nymphs							
Fed (%)		100	84.6	100	100	97.5	97.2
Molted (%)		100	9.1	16.3	17.8	87.2	97.1
Mortality (%)		0	92.3	83.7	82.2	15.0	5.6
Females							
Fed (%)		100	66.7	88.9	80.0	70.0	90.0
Fertile (%) ^4^		100	50.0	87.5	87.5	85.7	55.6
Unfed+infertile (%) ^5^		0	66.7	22.2	30.0	40.0	50.0

^1^ Percentage of fed ticks recovered from immunized mice; ^2^ percentage of molted ticks within the fed ticks recovered; ^3^ overall mortality during or after feeding; ^4^ percentage of fed females that laid eggs; ^5^ percentage of unfed females and fed but infertile females.

**Table 2:** Vaccine efficacy based on mortality, development, and reproductive parameters in larvae, nymphs, and females fed on mice previously immunized with the rAs8.9kDa (8.9), rAsBasicTail (BT), rAsKunitz (Kn), and rAsChimera (Ch) combinations.

Groups ^1^	RL	VL	RN	VN	RA	OA	FE	E (%) ^2^
Ct (Control)	1.00	1.00	1.00	1.00	1.00	1.00	1.00	-
V1 (8.9Ch)	0.68	0.14	0.85	0.09	0.67	0.55	0.26	99.9
V2 (BtCh)	0.73	0.17	1.00	0.16	0.89	0.84	0.56	99.2
V3 (KnCh)	0.84	0.18	1.00	0.18	0.80	0.51	0.76	99.2
V4 (Kn8.9Ch)	0.96	0.53	0.98	0.87	0.70	0.72	0.38	91.7
V5 (KnBt8.9Ch)	0.99	0.63	0.97	0.97	0.90	0.46	0.75	81.6

^1^ Experimental groups (antigen combination); ^2^ vaccine efficacy (E) was calculated according to Aguirre et al. [26], where E(%) = 100 × [1 − (RL × VL × RN × VN × RA × OA × FE)], and RL is the effect of vaccination on larvae recovery = RLvaccinated/RLcontrol; RN is the effect of vaccination on nymph recovery = RNv/RNc; RA is the effect of vaccination on adult female recovery = RAv/Rac; VL is the effect on larvae viability = VLv/VLc; VN is the effect on nymph viability = VNv/VNc; OA is the effect on female oviposition, which is the ratio between the average weight of the egg mass laid by females in the vaccinated (OAv) and control (OAc) groups = OAv/OAc; and FE is the effect on egg fertility, which is the ratio between the average hatchability (%) of eggs laid by females in the vaccinated (FEv) and control (FEc) groups = FEv/FEc.

## Data Availability

Data generated during the current study are available upon request to the corresponding author.

## Supplementary Materials

The following supporting information can be downloaded at: https://www.mdpi.com/article/10.3390/vaccines13020136/s1, Figure S1. Representative dot plots illustrating the immunophenotyping analysis in peripheral blood from mice; Table S1. Kidney and liver function of mice immunized with rAs8.9kDa (8.9), rAsBasicTail (BT), rAsKunitz (Kn), and rAsChimera (Ch) combinations; Table S2. Hematological parameters of mice immunized with rAs8.9kDa (8.9), rAsBasicTail (BT), rAsKunitz (Kn), and rAsChimera (Ch) combinations.
