# Supplementary material for: Immunoprotection Provided by Salivary and Intestinal Protein-Based Antigens Against the Ixodid Tick Amblyomma sculptum
Source: Vaccines (Basel). 2025 Jan 28;13(2):136. doi: 10.3390/vaccines13020136 (PMC11860927; doi:10.3390/vaccines13020136)
Supplement: Supplementary file 1 [file vaccines-13-00136-s001.zip › vaccines-3381510-supplementary.pdf]

Supplementary material

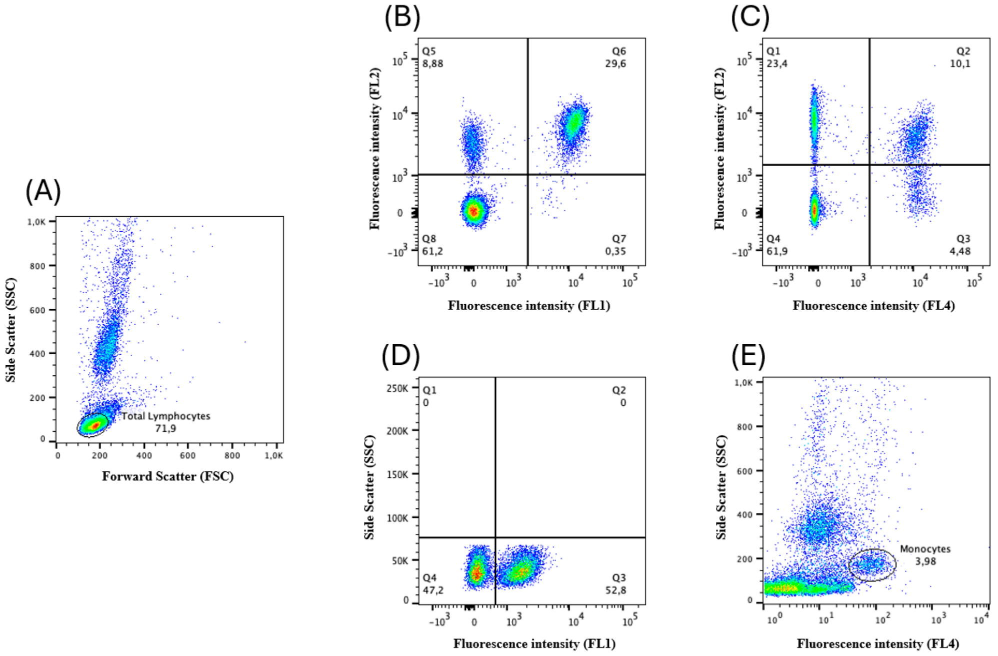

Figure S1. Representative dot plots illustrating the immunophenotyping analysis in peripheral blood from mice. CD4 T cells, CD8 T cells, B cells, and monocytes were characterized by flow cytometry using the following antibodies: anti-CD3 PE (FL2), anti-CD4 FITC (FL1), anti-CD8 APC (FL4), anti-CD19 FITC (FL1) and anti-CD14 APC (FL4). (A) Total lymphocytes were gated based on low size and granularity (FSC vs SSC). CD4 and CD8 T cells were selected as CD3+CD4+ (B) and CD3+CD8+ (C) double-positive populations within total leukocytes. B cells were identified as CD19+ cells within total leukocytes (D). Monocytes were selected based on CD14+ expression and intermediate granularity (E).

Table S1. Kidney and liver function of mice immunized with rAs8.9kDa (8.9), rAsBasicTail (BT), rAsKunitz (Kn) and rAsChimera (Ch) combinations.

Parameter	Urea (mg/dL)		ALT(U/L)[1]		AST(U/L)[2]	
Experimental Day	D-2	D40	D-2	D40	D-2	D40
Ct - Control	62.4 ± 4.6	63.3 ± 4.8	76.0 ± 7.2	77.8 ± 6.0	94.1 ± 11.6	124.0 ± 9.5
V1 - 8.9Ch	65.5 ± 3.7	65.3 ± 3.9	61.4 ± 4.8	67.0 ± 3.8	81.6 ± 5.6	141.8 ± 11.3**
V2 - BtCh	62.4 ± 2.6	63.0 ± 3.6	53.9 ± 4.0	55.8 ± 4.6	83.6 ± 11.5	121.7 ± 5.4*
V3 - KnCh	58.9 ± 6.0	59.0 ± 6.2	72.1 ± 6.5	72.9 ± 5.9	139.6 ± 10.6	139.4 ± 10.7
V4 - Kn8.9Ch	54.5 ± 13.2	63.6 ± 5.4	57.6 ± 10.4	64.7 ± 4.0	124.6 ± 24.4	155.8 ± 14.1
V5- KnBt8.9Ch	45.7 ± 7.0	52.5 ± 2.3	46.4 ± 3.8	47.3 ± 2.8	86.6 ± 7.4	116.3 ± 8.7*

[1]ALT - alanine aminotransferase; [2]AST - aspartate aminotransferase; Data is shown as mean ± SD; Blood from five animals of each group were used in the analysis; Statistical analysis: the T test was used to detect differences between D-2 and D40, *p<0.05, **p<0.01.

Table S2. Hematological parameters of mice immunized with rAs8.9kDa (8.9), rAsBasicTail (BT), rAsKunitz (Kn) and rAsChimera (Ch) combinations.

Parameter	Eritrocytes ($x10^6/\mu L$)		Platelets ($x10^3/\mu L$)		Hemoglobin (g/dL)		MCH[1] (pg)		MCV[2] (fL)		MCHC[3] (g/dL)		Leucocytes ($x10^3/mm^3$)	
Experimental day	D-2	D40	D-2	D40	D-2	D40	D-2	D40	D-2	D40	D-2	D40	D-2	D40
Ct - Control	8.1 ± 0.4	8.6 ± 0.1	1.5 ± 0.3	1.3 ± 0.1	14.2 ± 0.5	14.7 ± 0.2	17.6 ± 0.3	17.4 ± 0.3	53.4 ± 2.5	56.5 ± 0.9	33.0 ± 1.1	30.6 ± 0.4	5.8 ± 0.6	11.6 ± 6.2
V1 - 8.9Ch	8.3 ± 0.1	8.7 ± 0.1	1.3 ± 0.3	1.4 ± 0.2	14.3 ± 0.3	14.8 ± 0.2	17.2 ± 0.5	17.0 ± 0.3	54.4 ± 2.3	56.8 ± 1.4	31.8 ± 1.5	30.0 ± 0.4	5.9 ± 0.6	7.9 ± 1.5
V2 - BtCh	8.3 ± 0.1	8.3 ± 0.2	1.4 ± 0.3	1.2 ± 0.2	14.5 ± 0.2	14.5 ± 0.7	17.6 ± 0.3	17.5 ± 0.5	57.2 ± 2.8	59.9 ± 1.9	29.1 ± 0.5	30.8 ± 1.6	6.7 ± 0.6	8.6 ± 1.3
V3 - KnCh	8.4 ± 0.1	8.8 ± 0.2	1.9 ± 0.4	1.3 ± 0.1	14.7 ± 0.4	15.1 ± 0.5	17.5 ± 0.2	17.1 ± 0.3	49.7 ± 0.1	56.1 ± 0.5	35.3 ± 0.3	30.5 ± 0.2*	7.9 ± 2.9	9.5 ± 1.7
V4 - Kn8.9Ch	7.1 ± 0.5	8.7 ± 0.3	1.1 ± 0.2	1.5 ± 0.3	13.2 ± 1.4	15.0 ± 0.4	16.2 ± 0.5	17.2 ± 0.2	55.2 ± 0.9	57.5 ± 1.6	29.1 ± 1.3	31.2 ± 1.3	7.7 ± 0.4	7.9 ± 0.4
V5- KnBt8.9Ch	8.7 ± 0.2	8.8 ± 0.2	1.3 ± 0.1	1.3 ± 0.1	15.5 ± 0.2	15.4 ± 0.1	17.9 ± 0.3	17.6 ± 02	55.6 ± 2.1	57.6 ± 0.9	32.4 ± 1.1	30.6 ± 0.6	7.9 ± 1.4	8.8 ± 1.1

[1] HCM - Mean corpuscular hemoglobin; [2] MCV- Mean corpuscular volume; [3] MCHC - Mean corpuscular hemoglobin concentration; Data is shown as mean ± SD; Blood from five animals of each group were used in the analysis; Statistical analysis: the T test was used to detect differences between D-2 and D40, *$p<0.05$.
